# Electron beam surface remelting enhanced corrosion resistance of additively manufactured Ti-6Al-4V as a potential in-situ re-finishing technique

**DOI:** 10.1038/s41598-022-14907-2

**Published:** 2022-07-08

**Authors:** Mohammadali Shahsavari, Amin Imani, Andaman Setavoraphan, Rebecca Filardo Schaller, Edouard Asselin

**Affiliations:** 1 Department of Materials Engineering, The University of British Columbia, Vancouver, BC V6T 1Z4 Canada; 2 Sandia National Laboratories, Albuquerque, NM USA

**Keywords:** Engineering, Materials science

## Abstract

This study explores the effect of surface re-finishing on the corrosion behavior of electron beam manufactured (EBM) Ti-G5 (Ti-6Al-4V), including the novel application of an electron beam surface remelting (EBSR) technique. Specifically, the relationship between material surface roughness and corrosion resistance was examined. Surface roughness was tested in the as-printed (AP), mechanically polished (MP), and EBSR states and compared to wrought (WR) counterparts. Electrochemical measurements were performed in chloride-containing media. It was observed that surface roughness, rather than differences in the underlying microstructure, played a more significant role in the general corrosion resistance in the environment explored here. While both MP and EBSR methods reduced surface roughness and enhanced corrosion resistance, mechanical polishing has many known limitations. The EBSR process explored herein demonstrated positive preliminary results. The surface roughness (R_a_) of the EBM-AP material was considerably reduced by 82%. Additionally, the measured corrosion current density in 0.6 M NaCl for the EBSR sample is 0.05 µA cm^−2^, five times less than the value obtained for the EBM-AP specimen (0.26 µA cm^−2^).

## Introduction

Due to the excellent properties of titanium and its alloys, such as high corrosion resistance, high strength to weight ratio, and biocompatibility, they have shown great potential for a wide range of applications, including aerospace, automotive, marine, energy, and medical implant industries^1–5^. Their high corrosion resistance is due to forming a thin protective passive layer, a natural passive oxide that provides biocompatibility and strong resistance to pitting corrosion^6^. However, titanium has shown corrosion susceptibility when a break or defect in this oxide leads to localized corrosion, including pitting corrosion^6–8^. Electron beam melting (EBM), as a subset of powder bed fusion additive manufacturing (AM), is a new technology able to fabricate near-net-shape and tailor-designed parts with small tolerances and great geometrical flexibility. During EBM processing, parts are manufactured layer by layer under vacuum; a high-energy electron beam melts and solidifies powder in a pre-programmed pattern until the final desired model is made. Due to the increased attention to EBM manufactured Ti-G5 as a promising alloy for broad applications, extensive research has been carried out on its microstructural and mechanical properties^1,3,9–15^. Some comparison has been made of selectively laser melted (SLM) alloys with EBM materials^7,16–18^. In general, EBM Ti-G5 parts exhibited a rougher surface compared to SLM samples^7,16,17^ and comparable mechanical properties to the wrought (WR) alloy^10^.

AM surface roughness can be classified into two types; “primary roughness” and “secondary roughness,” in which the roughness is generated through melt pool solidification or partially melted powder particles, respectively^19^. In addition to its sizeable diffusible spot^20^, the large powder particles in EBM could hinder the repelling of negatively charged particles, leading to a rougher surface^21^. The average particle size used in SLM is in the range of 10–60 µm in diameter, whereas, for EBM, this varies in the range of 50–150 µm. Also, the high scan speed in EBM (more than 1000 m s^−1^) can cause increased adherence of larger powders to the surface^22^. Other typical surface defects contributing to increased surface roughness of EBM samples are the “stairstep effect,” “balling,” and “satellite”^7, 17, 22,23^. Because of the low and irregular amount of matter that a rough surface contains, it is considered mechanically inefficient^24^. This means that a rough surface can negatively contribute to tribological behavior and part tolerance. The final surface quality is influenced by the type of equipment, the direction of the build, and the process parameters used^25^. One of the limitations of EBM for industrial applications is that as-fabricated parts have a rough surface leading to possibly unfavorable corrosion properties^6^. It has been shown that uneven and rough surfaces may reduce the corrosion resistance of alloys^6,7^. Irregularities on the surface can act as potential “crevice formers” and/or sites for the initiation of pitting corrosion^26,27^. Additionally, it has been observed for EBM parts that defects and voids have adverse effects on the protective oxide layer and can also reduce the overall corrosion resistance^8^. For example, potentiostatic testing (for Ti-G5 in chloride-containing solution at 800 mV_SCE_) exhibited a lower critical pitting temperature for higher surface roughness EBM samples^8^. However, In some specific applications, surface roughness is desirable; for example, in biomedical applications, surface roughness can assist tissue growth and adherence to implant surfaces^28^. Thus, because of different impacts of surface roughness on material properties and their applications, a better understanding of the governing factors for AM surface finish and subsequent material behavior is essential before application.

As surface finish has been established as a significant parameter affecting as-built AM material properties, an increased interest has grown in the literature exploring the effects of EBM processing parameters on surface roughness^29–31^. A rough surface and insufficient surface quality can also be improved by different post-process surface finishing methods such as mechanical (sandblasting^32^, abrasive blasting^27^), chemical (acid etching^33^, oxidation), electrochemical (passivation, electropolishing^34^), thermal processes (micro-arc oxidation^35^), and laser treatment^36^. Some authors reported the application of the laser surface treatment technique for AM-produced parts without detachment from the build plate^37–39^. However, ex-situ laser treatment was studied as an effective method to decrease the surface roughness of the EBM parts by 80%^36^. On the other hand, laser surface remelting (LSR) was reported as an effective in-situ method to enhance the surface roughness of SLM-produced parts^37–39^. While LSR was shown as a potential in-situ surface treatment method within the SLM process, this work tries to address the effectiveness of EBSR, which can be applied either in or ex-situ, with the aid of an e-beam welder, as a possible solution to enhance the surface quality. For in-situ EBSR, the technique can be applied either layer by layer or solely for the final surface finish (last layer).

In this study, EBSR was employed as an effective way to enhance the surface roughness and subsequent corrosion resistance of EBM Ti-G5 material. Corrosion properties of EBM vs. traditional WR counterparts of Ti-G5 were evaluated through electrochemical analysis in 0.6 M NaCl solution. In addition, the effects of various surface finish methods on surface roughness and subsequent corrosion resistance for EBM and WR samples were investigated. The WR and EBM samples in as-printed (AP), mechanically polished (MP), and EBSR states were studied to examine the effects of EBSR process on the surface roughness and corrosion resistance. Microstructural and phase characterization was carried out using X-ray diffraction (XRD), Field Emission Scanning Electron Microscopy (FE-SEM), Energy Dispersive Spectroscopy (EDS), and Optical Microscopy (OM) for additional comparison.

## Results

### Microstructural and phase characterization

**Figure 1:**
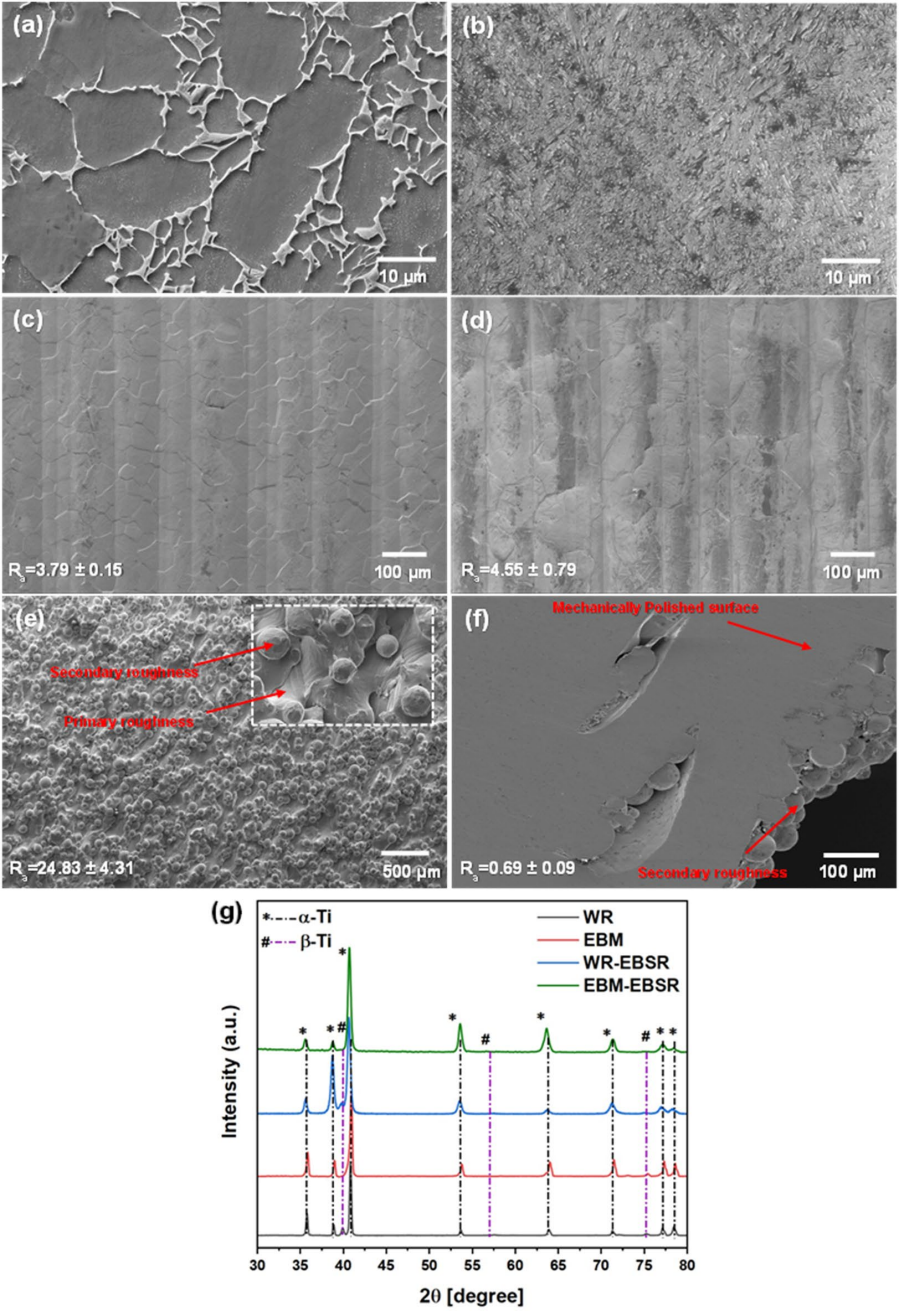
FE-SEM micrographs of (**a**) WR and (**b**) EBM-AP Ti-G5 alloy. The α and β phases are shown by dark and light areas, respectively. Microstructures after EBSR on (**c**) WR and (**d**) EBM samples. The parallel lines show the scan track of the electron beam during the remelting process. (**e**) Un-melted and partially sintered particles on the as-printed surface, (**f**) lack of fusion defects and bulk pores on the as-printed surface after mechanical polishing. (**g**) XRD patterns of the WR and EBM materials before and after EBSR treatment.

Figure 1 shows the micrographs of WR and EBM-AP Ti-G5 samples. The WR microstructure in Fig. 1a consists of roughly equiaxed α grains (dark grey) with intergranular β (light grey). This WR microstructure may have evolved due to production or subsequent annealing; however, it is consistent with a typical microstructure for an annealed Ti-G5 ingot^40,41^. As shown in Fig. 1b, the microstructure of the EBM-AP sample consists of α and β phases, similar to the WR material. However, the morphology is very different; the α-grain boundary defines the columnar prior β grains for the EBM-AP sample. Some singular α bulges can be seen sandwiching the prior β grains, similar to^42^. Moreover, a dot-like morphology is observed at the β phase. Using ImageJ software (version 1.53 k, imagej.nih.gov/ij/)^43^, the WR equiaxed α-grains were found to be, on average, 20 ± 0.59 µm in size (300 grains). Intergranular β of the WR sample was also measured and was found to be on average 0.4 ± 0.03 µm in width. The average width of the lamellar α phase was measured around 0.6 ± 0.04 µm and 1.6 ± 0.08 µm for EBM-AP and WR, respectively. Hence, the EBM-AP consists of finer lamellar α and β phases and possesses more grain boundaries than the WR counterpart. This is likely due to the rapid cooling rate during the EBM process^1,2,4^. Figure 1c,d show the plan view images displaying the surface of WR and EBM samples, respectively, after the EBSR process. Figure 1e shows un-melted particles on the AP surface, which could be classified as “balling” or “satellite” defects^23^. These particles, which result from either spreading particles from the melt pool due to the high flow rate or lack of fusion particles, can lead to high levels of surface perturbation. Partially sintered particles and the defect caused by lack of fusion are clearly shown in Fig. 1f. Two types of surface roughness, i.e., the “primary” and “secondary”, are shown in Fig. 1e,f. The former relates to the roughness induced by the solidification of the melt pool, while the latter is attributed to the unmelted or partially melted particles^44^.

Figure 1g shows the XRD patterns of the WR and EBM samples before and after EBSR treatment. A typical XRD profile for α/β Ti-G5 material is observed. It is known that that the differentiation of the α phase and martensitic α’ phase is difficult because they have the same HCP crystal structure and similar lattice parameters for these two phases^18,42,45,46^. However, in comparison to the SLM-fabricated Ti-G5 parts, it is less likely to observe the α’ phase in EBM materials as the cooling rate in the latter is much lower^18,42,46,47^. An important observation from the XRD patterns is that the EBSR process did not seem to alter the phase composition of the materials.

### Surface characterization

**Figure 2:**
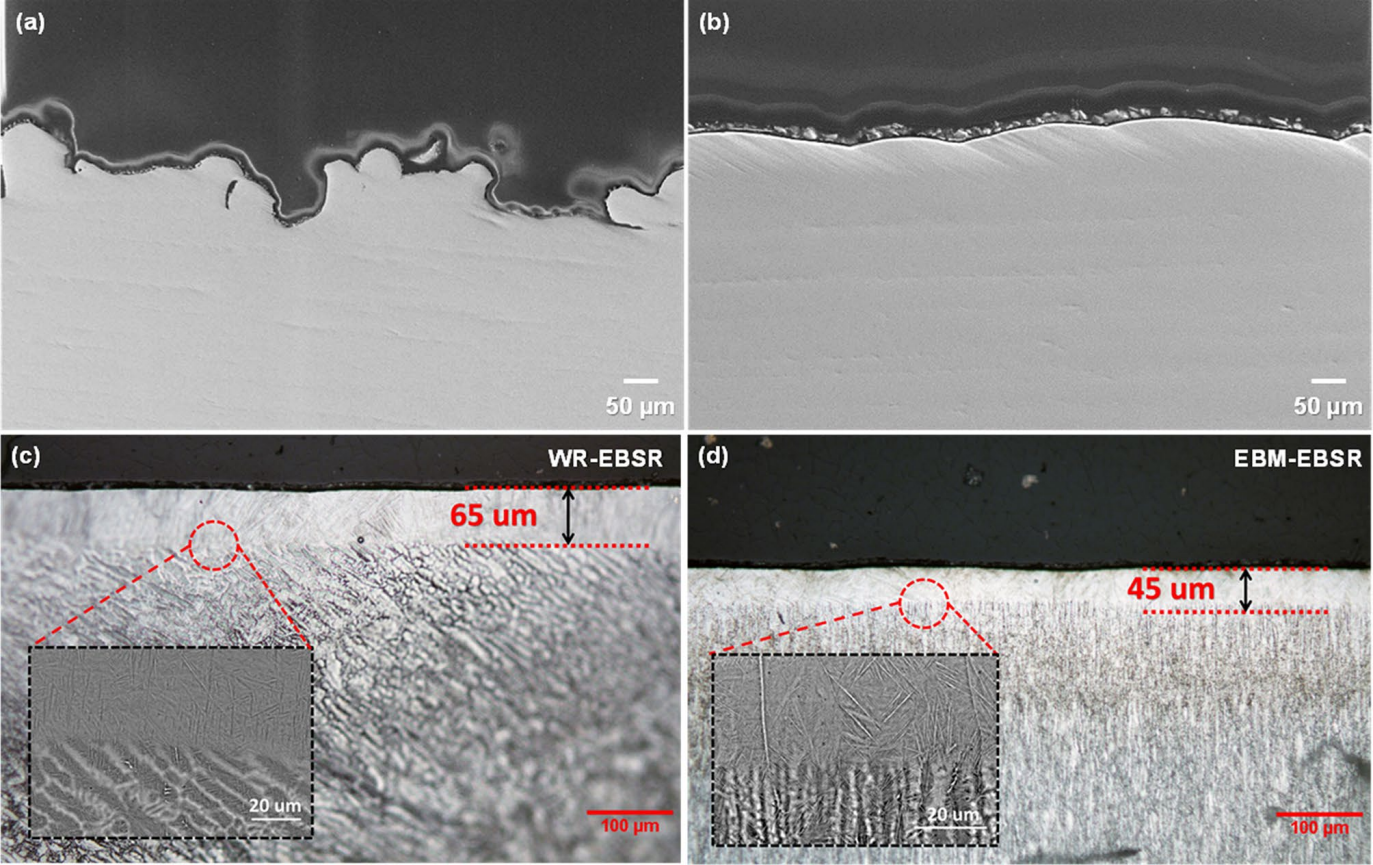
Cross-sectional FE-SEM (**a**,**b**) and OM (**c**,**d**) images of EBM-AP (**a**), EBM-EBSR (**b**), WR-EBSR (**c**), and EBM-EBSR (**d**). The samples in (**c**) and (**d**) were etched and the insets show the SEM micrographs.

Table 1 lists the roughness values for AP-, EBSR-, and MP- EBM specimens. The AP surface of the EBM-produced Ti-G5 displayed the highest roughness value. Post-processing techniques, such as MP, were shown to reduce the surface roughness by two orders of magnitude (from 24.83 ± 4.31 µm in AP to 0.69 ± 0.09 µm for MP). Figure 1f shows an SEM image after polishing with SiC paper to 1200 grit finish. It seems that the MP was unable to remove all the porosities as some of the pores were located deeper from the surface. Therefore, removing the top layer of unevenness by MP did not eliminate the deeper pores in the sample. The observed pores in Fig. 1f are likely due to either argon gas or water vapor entrapment^21, 48,49^. Initial results confirmed that EBSR is a potential in-situ technique that reduces surface roughness. Here, initial measurements displayed a reduction in surface roughness from 24.83 ± 4.31 µm for the AP sample to 4.55 ± 0.79 µm in EBSR (82% improvement in surface roughness from AP state). Figure 2 displays secondary electron SEM images of cross-sections and compares the surfaces before and after EBSR on the EBM sample for better visualization. This indicates the apparent effects of EBSR on surface roughness with reduced unevenness, reduction of possible crevice formers, and smooth overall appearance as compared to the AP counterpart. A significant change in surface roughness by EBSR (82% improvement) is evident.

**Table 1:** Surface roughness values for EBM and WR samples in different surface states of as-printed (AP), electron beam surface remelted (EBSR), and mechanically polished (MP).

Surface condition	R_a_ (µm)
EBM as-printed	24.83 ± 4.31
EBM- electron beam surface remelted	4.55 ± 0.79
EBM- mechanically polished (1200 grit)	0.69 ± 0.09
WR- electron beam surface remelted	3.79 ± 0.15

The mechanical polishing on the WR sample would result in a similar surface roughness to EBM as the same sandpaper (1200 grit) was used for both samples.

The cross-sections of the WR and EBM samples after EBSR treatment were etched and examined by SEM and OM to identify the thickness of the re-melted layer and potential microstructural alterations. Figure 2c,d illustrate the etched cross-sectional OM and SEM (inset) images of the WR and EBM materials after EBSR treatment. It was observed that the thickness of the EBSR layer was about 65 and 45 µm for the WR and EBM samples, respectively. The SEM images revealed that the EBSR treatment changed the microstructure of the WR sample from equiaxed to elongated α grains with intergranular β phases. Looking at the microstructure of the WR sample at distances beyond ca. 65 µm from the outermost EBSR layer confirms that the bulk morphology remained intact with the presence of the equiaxed α grains, similar to the untreated WR material (Fig. 1a). On the other hand, the microstructure of the EBM sample did not seem to have changed significantly by the EBSR treatment; the columnar prior β grains are defined by the α-grains, leaving a similar structure to the WR-EBSR counterpart. Similar alterations in microstructure after post processing to enhance the surface roughness have also been observed for laser treated EBM samples^36,50,51^.

### Electrochemical measurements

**Figure 3:**
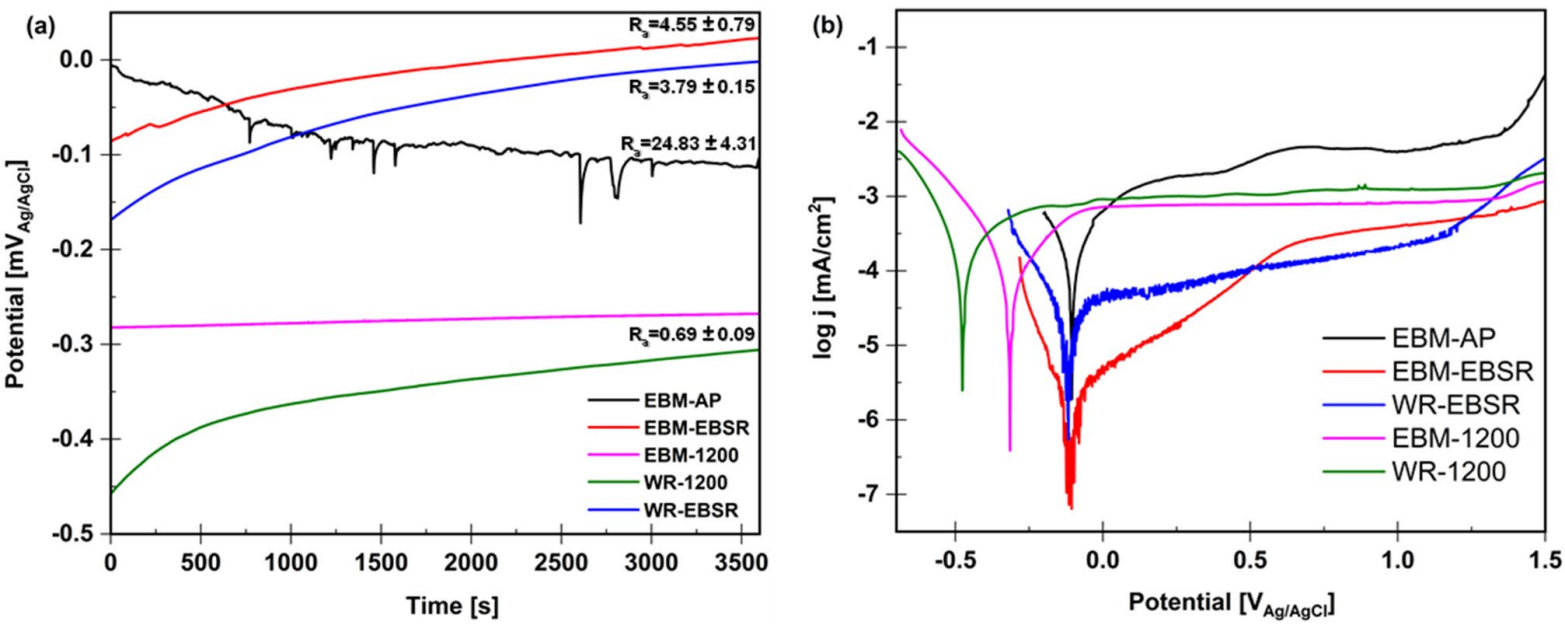
(**a**) OCP and (**b**) PDP curves of the EBSR, EBM, and WR samples with different surface roughness values in 0.6 M NaCl solution.

Figure 3a illustrates the open circuit potential (OCP) trends in 0.6 M NaCl for EBSR, EBM, and WR samples with different levels of surface roughness. An increase in the potential over time is recognized for all samples except for the EBM-AP. The increasing trend and then stabilization of OCP for EBM-MP, EBSR, and WR materials are consistent with forming a passive protective layer on the surface and, consequently, improved passive behavior^52,53^. Although the OCP for the EBM-AP decreased over time, it tended to stabilize at longer times, indicating the formation of a passive protective layer^34^. Some fluctuations were observed for the EBM-AP sample, implying the instantaneous competition between passive film formation and metal dissolution^42,54^. However, after immersion for roughly 3000 s, the OCP stabilized at around − 0.1 V_Ag/AgCl_, denoting the formation of the stable passive film in 0.6 M NaCl. The initial drop in the OCP of the EBM-AP is likely due to the heterogeneities in the surface, which passivate over time. The most positive OCP, recorded for the EBSR sample, might signify the formation of a more protective passive layer compared to other surface finish methods. The more negative OCP values for WR vs. other specimens suggest that the WR sample has a thermodynamically higher tendency for corrosion in 0.6 M NaCl^55,56^.

The potentiodynamic polarization (PDP) curves in 0.6 M NaCl are shown in Fig. 3b. Table 2 summarizes the corrosion parameters, including the open circuit potential (E_OCP_), corrosion potential (E_corr_), corrosion current density (j_corr_), cathodic Tafel slope (β_c_), and passivation current density (j_p_). As all samples displayed the typical passive behavior of a Ti alloy, the cathodic Tafel slope was used to determine the corrosion parameters^42,57,58^. According to PDP curves in Fig. 3b and corrosion parameters in Table 2, with decreasing surface roughness, E_corr_ decreased towards more negative values for both EBM and WR samples after MP and EBSR, indicating an increase in the electrochemical surface activity for corrosion, as the oxidation reaction is more likely to happen^59^. In addition, since all samples showed a typical passive behavior of a titanium alloy, the slight differences in corrosion potentials for EBM-AP, EBM-EBSR, and WR-EBSR are indicative of the surface state, and not necessarily the corrosion kinetics. The j_corr_ parameter can be used to better compare between the surface roughness and corrosion rate of the tested samples. The j_corr_ values decreased with decreasing roughness, implying a reduction in the corrosion rate as the surfaces of both EBM and WR become smoother. Furthermore, j_corr_ for EBM-1200 after grinding is much lower than the WR sample, confirming the lower corrosion rate for the EBM-1200 sample. Likewise, as shown in Fig. 3b and Table 2, j_corr_ value decreased from 0.26 µA cm^−2^ for EBM-AP to 0.05 µA cm^−2^ for EBM-EBSR, which is the lowest j_corr_ value amongst all samples. Likewise, it could be seen that the EBSR treatment on the WR sample resulted in the lowest j_corr_ of 0.11 µA cm^−2^ among other WR materials. The decreasing j_corr_ trend for EBSR treated samples confirms the effectiveness of this technique in decreasing the corrosion rate, particularly for the EBM-AP material.

**Table 2:** Corrosion parameters obtained in 0.6 M NaCl for EBM and WR samples after different post-processing methods.

Surface condition	E_OCP_ at 3600 s (mV_Ag/AgCl_)	E_corr_ (mV_Ag/AgCl_)	j_corr_ (µA cm^−2^)	β_c_ (mV/decade)	j_p_ (µA cm^−2^)
EBM-AP	− 115	− 104	0.26	241	1.9
EBM-EBSR	22	− 114	0.05	112	0.3
WR-EBSR	2	− 117	0.11	124	0.1
EBM–MP 1200	− 268	− 314	0.13	225	0.8
WR–MP 1200	− 306	− 475	0.21	275	1.1

According to Table 2, j_p_ values slightly dropped with decreasing surface roughness by mechanical polishing. The slightly lower j_p_ for the EBM-1200 than WR-1200 means that the passivation of the former was more accessible, and its stability and protection were comparable to that of the latter^42,46^. Also, the j_p_ values of 0.3 and 0.1 µA cm^−2^ for EBM- and WR-EBSR, respectively, suggest the formation of more protective passive layers on the EBSR treated surfaces, leading to their enhanced corrosion resistance. A comparison of all PDP curves reveals that decreasing the surface roughness, independent of the material, facilitates the formation of the passive film, thus improving the corrosion resistance. In other words, where only the effect of surface roughness is considered, the difference in microstructures of the EBM and WR materials did not play a significant role in the corrosion resistance in 0.6 M NaCl. This was confirmed by the similar corrosion behaviors of mechanically polished EBM and WR, particularly in the passive region. Overall, a comparison of OCP trends and PDP curves for the WR, EBM-AP, WR-EBSR and EBM-EBSR displayed a considerable enhancement for the EBSR-treated samples. It was observed that although the microstructures of both EBSR and EBM-1200 were the same, the former’s corrosion resistance was considerably enhanced as a result of EBSR. This implies an important effect of surface roughness on corrosion behavior compared to possible influences of the underlying microstructure.

## Discussion

### Effect of EBM process on microstructure and corrosion resistance

Results showed that where the surface roughness of EBM and WR materials are identical, the corrosion resistance of the former is slightly enhanced. The lower j_p_ and j_corr_ confirmed this in conjunction with a more positive E_OCP_ for the EBM sample. We have shown in our recent study that the slightly better corrosion resistance of the EBM material than WR is due to a higher amount of β phase in its microstructure^45^. The different nature of the EBM process enhances the resultant microstructure by forming more β phase due to the faster cooling rate. It was previously shown that the melt pool cooling rate is much higher than that of the already-printed sections of the sample in an EBM process^18, 41^. Decreasing the temperature from the melting point of Ti-G5 (i.e., 1600 °C) to the substrate temperature (about 400–500 °C) within a short time in the vacuum chamber results in a faster cooling rate than the conventional Ti-G5 casting (WR alloy product). When the temperature falls below the β transus line, the high temperature β phase having a BCC lattice structure transforms to a more stable α phase with the HCP structure. The cooling rate critically determines the amount of transformed β to α^42,60^. More importantly, the rapid cooling rate would hinder the β to α transformation so, under a fast cooling rate, the final amount of β is higher. The higher fraction of the β phase could lead to enhanced corrosion resistance by increasing the charge transfer resistance through the double layer and reducing the rate of the dissolution reaction^42,45^. Vanadium (V) is a β phase stabilizer, and aluminum (Al) is an α phase stabilizer. The β phase enriched in V plays a vital role in improving the corrosion resistance of the EBM alloy. Higher V content in the β phase results in higher resistance against dissolution since the β phase is more protective against selective corrosion than the α phase^42,61^. Moreover, the fine α and β grains present in the microstructure of the EBM sample might contribute to its superior corrosion resistance compared to the coarser α phase in the WR sample^45,55,62^. From the results of this study, the differences in the microstructure of the WR and EBM due to their manufacturing methods suggest that these materials will possess slightly different corrosion properties. The microstructure difference seems to become important when the surface roughness values are identical.

### Effect of microstructure and surface roughness on corrosion resistance and the importance of surface finish

It is known that the microstructure and surface finish are two important factors affecting the corrosion properties of materials^45,63^. In terms of microstructural features, it has been widely shown that the larger amount of β phase in Ti-G5 alloys, compared to the α phase, improves the corrosion resistance of the material^42,45,64,65^. This has been explained by the formation of a more protective passive film on the β phase due to the higher vanadium content as the phase stabilizer^42,45,64,65^. Additionally, it was reported that the α phase has inferior corrosion resistance because its corrosion rate at the α/β interface at OCP was higher than the β phase^66^. For AM parts, however, the presence of the martensitic α’ phase could result in the formation of a less stable passive film due to the depletion of aluminum and vanadium in this non-equilibrium phase^52,65,67^. It would be difficult to distinguish the α and α’ phases in XRD patterns as they both have an HCP crystal structure with similar lattice parameters^18,42,45,46^. It is also claimed that the formation of the α’ phase in EBM-fabricated materials is unlike that found in SLM-printed samples. This is attributed to the much lower cooling rate in the EBM process^18,42,46,47^. Moreover, no needle-like martensitic α’ structure could be detected in the OM images of the untreated and EBSR-treated WR and EBM materials after etching^68,69^. The OM images of the same materials as studied herein have been provided elsewhere^69^. Therefore, it could be concluded that the α’ martensite was not present in the untreated and EBSR-treated WR and EBM materials. However, more evaluations using higher resolution techniques are necessary to confirm. The SEM images also revealed the difference in the size of microstructural features as the EBM material had a finer microstructure than the WR, which has larger equiaxed grains. It was shown that a finer microstructure of the EBM Ti-G5 promoted the formation of the passive film due to the presence of higher active sites for the nucleation and growth of the passive film^42,46^. Therefore, the possibility of the formation of a galvanic cell between the grains due to the micro-segregation of the alloying elements is decreased, thus increasing corrosion resistance^42,46,70^.

While both the microstructure and surface morphology influenced the corrosion response of the specimens examined herein, surface roughness played a more significant role in the corrosion response of the WR and EBM alloys than the microstructure. Decreasing the roughness significantly improved corrosion protection in the samples. In comparison, the effect of WR vs. EBM microstructure plays an essential role for samples with similar surface finish and may govern corrosion resistance at the local scale. Future work may evaluate the synergistic effect of surface roughness and microstructure on the corrosion resistance of the WR and EBM materials after EBSR. One of the limitations of the EBM process is the poor surface quality of fabricated parts, which can negatively influence the corrosion resistance. As observed from experimental data and microstructural analysis, EBM as-fabricated parts have a rough surface due to the large spot size, large powder particles, and high scan speed^19,21,22,28,29^. These factors can leave unmelted particles on the final surface, creating various defects such as “balling” or “satellites.” Consequently, these defects result in high surface perturbation levels. The rough surface may contribute to the presence of crevice formers^7,8,26^ and micro-pits (related to micron-sized defects)^42^, which could all increase corrosion. The rough surface of as-printed EBM parts could lead to the breakdown of the titanium passivity over a crevice former or pit^7,8,26^. The effect of surface roughness on the corrosion behavior of the EBM sample is related to the heterogeneous surface, particularly the un-melted particles on the surface that act as initiation points and possible “crevice formers”, which exacerbate corrosion^7,8^. Therefore, these particles can enhance initiation and localized corrosion rates due to the complex geometries they create at the surface. Higher corrosion rates on rough WR and EBM materials could be described by the available active sites on the surface. On the other hand, better corrosion resistance in samples with lower surface roughness corresponds to the rapid formation of a stable passive film on the surface^71,72^.

Rough surfaces may not be appropriate for services where high corrosion resistance is required. However, as shown herein, surface finish methods can improve the poor surface quality and inferior corrosion behavior of rough EBM parts. Accordingly, post-processing methods might be applied during EBM or after manufacturing. Mechanical polishing is one of the surface finishing methods, which gives a final surface with an acceptable roughness level, as observed in this study. However, it has some drawbacks, making it inappropriate for many applications. These drawbacks include lack of dimensional accuracy and inability to remove all porosities^73^. In addition, other drawbacks of mechanical polishing, similar to physical machining processes, are their need for repeatability for better quality, the use of mechanical tools and their contact with the surface, which could result in parts deformation and interrupt the dimensional accuracy^36,50,74^. Moreover, other surface finishing methods, such as chemical etching, are not environmentally friendly due to the involvement of chemical agents and solvents that are harmful to the environment as well as human safety^33,36,50^. Physical machining processes, such as lathing, milling, grinding, etc., may also result in material wastage, deformation, and dimensional inaccuracy. Due to the mentioned limitations, mechanical polishing, physical machining, and chemical etching are not applicable in most cases, particularly for metallic biomaterials and parts that require high dimensional accuracy in service^33,36,50,73^. Thus, the development of in-situ or enhanced ex-situ surface finish methods for EBM-fabricated parts, such as EBSR, is necessary. On the other hand, similar to laser treatment methods, EBSR is a less chemically hazardous surface treatment technique with decreased material waste, no involvement of chemical agents and other materials like plastics^33,36,50^. Even though the higher cost may limit the use of laser and electron beams for a surface refinishing after the actual AM manufacturing, future research and development will focus on enhancing the availability and price of these methods as the AM industry is improving at a growing pace.

### Effects of EBSR process on the surface

The result of this study shows that the EBSR, as an in-situ post-processing technique, can reduce the surface roughness and enhance the corrosion resistance without many of the drawbacks of other post-processing surface treatments. Even though EBSR is not as simple as mechanical polishing, it can benefit the manufacturing industries to overcome the limitations of the latter. As the high-energy electron beam scans the surface during the EBSR process and re-melts the surface (see video file in SI), valleys could be filled in and decrease the number of pores^38^. The possibility of decreasing pores is highly dependent on the processing parameters, as proper remelting parameters could result in a high-quality surface with the lowest number of pores. On the other hand, unsuitable processing parameters could deteriorate the surface quality^38^. While laser surface remelting has achieved a 90% improvement in surface quality^37–39,75^, this work showed that the EBSR improved surface roughness by around 82%. However, the EDS results (Table 3) showed slight differences in composition across the surface. The ratios of Ti:Al and Ti:V were considered to interpret the EDS results. For both remelted surfaces, results showed an insignificant change in surface elemental compositions after EBSR (compared to the bulk composition) and a non-uniform elemental distribution at each surface as expected by Vaithilingam et al*.* for LSR^39^. During the EBSR process, surface chemical transformation, which depends on the alloying elements and post-processing build conditions, is probable while the surface roughness is improved^39,76^. According to the results, the amount of Al was a little bit higher than the standard amount of Al in Ti-G5. Likewise, the V content was slightly lower than the standard amount. This alteration in elemental composition due to the EBSR process could be attributed to the different processing parameters of EBSR compared to the actual fabrication of the sample. The machine used for EBSR in this study can impose different heating and cooling cycles to the remelted surface because of the different processing parameters, thus a slight difference in elemental composition. Alteration in elemental composition was also seen for laser surface remelting, and results showed a higher amount of Al and the lower amount of V on the treated surface after LSR^39^. The difference in elemental composition on the remelted surface compared to the bulk could be due to the rapid melting and solidification during the EBSR process, as mentioned for laser surface remelting^39^. In addition to elemental compositions of the remelted area, the microstructure of the EBSR layers of the WR and EBM samples were found to be altered, with the former having a more significant change. The electron beam welder machine used in this study for the EBSR process functions in a vacuum. Therefore, the presence of vacuum, which is like the EBM machine, seems to have resulted in a lower microstructural alteration for the EBM sample than the WR. This could have been achieved by a minimized contamination in the manufacturing process of reactive alloys such as Ti-G5^22,69,77,78^. Nevertheless, the exact role of different heating and cooling cycles in the electron beam welder machine requires further investigation.

**Table 3:** Chemical composition of post-EBSR samples by EDS (atomic%).

Element (wt%)	EBM-EBSR			WR-EBSR		
	Point 1	Point 2	Point 3	Point 1	Point 2	Point 3
Ti	85.69	85.32	85.18	85.19	85.71	85.48
Al	11.52	11.63	11.95	12.13	11.76	11.62
V	2.79	3.05	2.87	2.68	2.53	2.90

Our preliminary results show that the EBSR process is a promising technique that can decrease the surface roughness of the EBM-AP parts by 82%. EBSR, which can be applied either layer by layer or solely on the outermost surface, could benefit parts with complex geometry and high dimensional accuracy. However, more evaluations are required to shed light on the underlying mechanisms of re-melting and solidification that occur during this novel technique. Although this study provided results on the beneficial effect of electron beam surface remelting on the corrosion and electrochemical behavior of EBM Ti-G5, the impact of EBSR on other materials’ properties should also be examined. In addition, future work may consider implementing new technologies concerning the in-situ application of EBSR. That is, the surface roughness of the manufactured part can be enhanced without the need for the part removal from the chamber. Indeed, the EBM manufacturing cost and time are important considerations that need to be dealt with in future studies to assess their industrial feasibility on a large scale^74^.

## Conclusion

We evaluated the effect of surface roughness on the corrosion resistance of WR and EBM Ti-G5 materials with different microstructures and surface states. We also used EBSR as a novel in-situ method to improve surface roughness and corrosion resistance. Our results showed that:

- EBSR decreased surface roughness by 82% while increasing corrosion resistance.
- EBM-AP has the roughest surface due to the presence of un-melted particles on the surface. However, the surface quality was enhanced through EBSR and mechanical polishing to Ra = 4.55 ± 0.79 and 0.69 ± 0.09 µm, respectively.
- The lowest j_corr_ and j_p_ values obtained in 0.6 M NaCl for the EBSR sample showed the best corrosion resistance for the remelted surface.
- Nearly the same PDP curves for EBM and WR with similar roughness values signify that the microstructure dissimilarity is not the main reason for the difference in corrosion resistance. Thus, roughness mainly affects the corrosion resistance of EBM and WR Ti-G5 specimens in 0.6 M NaCl.

## Methods

The supporting information (SI) provides the details of the materials, electrochemical methods, microstructural and surface characterization. To investigate the effect of different surface finishes on the corrosion behavior of EBM and WR Ti-G5 alloys, two modes of surface finishing were performed: mechanical polishing (1200 grit) and EBSR. After each approach, the surface roughness of the EBM-AP, MP, and EBSR was measured via surface profilometer (Dektak XT, Bruker) controlled by Vision64 Operation and Analysis Software (Version 5.7, www.bruker.com) using random line scans with a length each of 4000 µm and a scanning time of 30 s. The stylus force was adjusted to 1 mg for all profilometry measurements, and the roughness values were obtained from the software. The performed surface finishing methods were as follows:

### Mechanical polishing

EBM and WR samples were ground to 1200 grit finish using SiC paper. Pieces were cleaned, rinsed with DI water, then ultrasonicated in acetone for 5 min, followed by air drying.

### Electron beam surface remelting

EBSR was performed on EBM, and WR samples for comparison using a modified Canmora Tech electron beam welder with the processing parameters listed in Table 4. This surface treatment was presented as a new method to enhance the surface roughness and mimic possible in-situ EBSR applications. Due to the nature of the current setup, EBSR was applied post build on the final surface, in vacuum, without the surrounding powder bed typical of EBM applications. However, future modifications and studies would include in-situ analysis of such a technique before application. The video showing the EBSR process can be found in supplementary files. The EBM-AP disc is exposed to a high-energy electron beam that scans the surface in vacuum.

**Table 4:** Electron beam surface remelted (EBSR) processing parameters.

Gun pressure (mbar)	3.57 × 10^−6^
Chamber pressure (mbar)	7.73 × 10^−5^
Accelerating voltage (kV)	90.3
Accelerating voltage limit (kV)	10
Laser current (mA)	Max 55
Beam current (mA)	7.01

Laser current was used to heat the cathode.

## Supplementary Information

Supplementary Video 1.

Supplementary Information 1.

## Data Availability

The datasets used and/or analysed during the current study available from the corresponding author on reasonable request.
