# Supplementary material for: Electron beam surface remelting enhanced corrosion resistance of additively manufactured Ti-6Al-4V as a potential in-situ re-finishing technique
Source: Sci Rep. 2022 Jul 8;12:11589. doi: 10.1038/s41598-022-14907-2 (PMC9270471; doi:10.1038/s41598-022-14907-2)
Supplement: Supplementary file 2 — Supplementary Information 1. [file 41598_2022_14907_MOESM2_ESM.docx]

**Electron beam surface remelting enhanced corrosion resistance of additively manufactured Ti-6Al-4V as a potential in-situ re-finishing technique**

Mohammadali Shahsavari^1^, Amin Imani^*1^, Andaman Setavoraphan^1^, Rebecca Filardo Schaller^1,2^ & Edouard Asselin^1^

^1^Department of Materials Engineering, The University of British Columbia, Vancouver, V6T 1Z4,

BC, Canada

^2^Sandia National Laboratories, Albuquerque, NM, USA

Corresponding author: [amin.imani@ubc.ca](mailto:amin.imani@ubc.ca)

**Materials**

Wrought (WR) and electron beam melted (EBM) Ti-G5 specimens were used in this study. The EBM disk samples were produced using an ARCAM A1 EBM Machine (from GPM2 group at the Université Grenoble Alpes in France), where the sample thickness was 1 mm, and the chamber He pressure was controlled at 2.10-3 mbar. The operating voltage of the machine was 60 kV accelerating. The thickness of each powder deposition layer was 50 µm on a stainless steel plate. The powder used was Ti-G5 extra low interstitial (ELI) metal powder, which falls under the ASTM 3001 specifications, with a spherical powder median diameter of 62 µm measured by SEM images. Argon gas was used in the atomization process for particle production. The samples used within were printed with the same material on the same machine as that used in ^1^. The compositional analysis of the Ti-G5 powder, provided by ARCAM AB, is given in Table S1. Circular discs with a diameter of 160 mm were printed parallel to the build direction. The WR billet with a diameter of 120 mm was from TIMET. Samples were received in an alpha-beta forged state and tested in the mill annealed condition. The WR specimens were cut into pieces with a diameter of 160 mm and a thickness of 2 mm using wire cutting.

**Microstructural, phase and surface characterization**

To investigate the microstructure of EBM and WR samples with different surface conditions, a CARL ZEIS SIGMA FE-SEM was used to take Field Emission Scanning Electron Microscopy (FE-SEM) images. A Nikon Epiphot 300 Inverted Microscope was used for optical microscopic (OM) images. Energy Dispersive Spectroscopy (EDS) was performed for elemental analysis after EBSR. SEM was used for the characterization of the as-built surface and microstructure without any prior heat treatment. The surface of the EBM-AP sample was imaged to observe the AP condition comprised of the rough surface and defects or un-melted particles. For microstructural analysis, WR and EBM-AP samples were ground to 1200 grit finish using silicon carbide paper followed by a mirror polish with 0.6 µm SiC slurry. Before characterization, all samples were cleaned by ultrasonication in acetone for 5 minutes to remove any surface contamination from grinding. Then, the mirror-like surface was etched in a Kroll’s reagent containing DI water, nitric acid, and hydrofluoric acid before being examined under SEM to observe the microstructures of the WR and EBM samples before and after EBSR. ImageJ software ^2^ was used to measure the lamellar grains of the EBM and WR samples. An average was taken from $50$ grain measurements. For analysis of the top surface pre– and post– EBSR, samples were cross-sectioned and mounted in epoxy. Prior to SEM characterization, a similar polishing procedure, as described above, was applied.

The phase composition of the WR and EBM Ti-G5 samples was evaluated using X-ray diffraction (XRD) by a Rigaku MultiFlex X-ray diffractometer with a 2 kW X-ray generator and a Cu-Kα radiation source.

| *Table S1. Elemental composition of Ti-6Al-4V powder under ASTM F3001 and EBM powder.* | | | | | | | | | |
| --- | --- | --- | --- | --- | --- | --- | --- | --- | --- |
| **Element (wt.%)** | **Al** | **V** | **C** | **Fe** | **O** | **N** | **H** | **Y** | **Ti** |
| ASTM F3001 | 5.50-6.50 | 3.50-4.50 | Max 0.08 | Max 0.25 | Max 0.13 | Max 0.05 | Max 0.012 | Max 0.005 | Bal. |
| EBM Ti-6Al-4V powder | 6.47 | 3.93 | 0.61 | 0.22 | 0.09 | 0.01 | 0.001 | <0.001 | Bal. |

**Electrochemical Analysis**

Both EBM and WR samples were tested in AP and ground conditions. The OCP tests were carried out for 3600 seconds to obtain a stable potential. After a stable OCP was achieved, PDP measurements were performed from −200 mV with respect to OCP to 2000 mV_Ag/AgCl_ with the scan rate of 0.166 mVS^–1^. All electrochemical tests were carried out in quiescent, 0.6M NaCl solution at ambient temperature. The measured pH was 6.8 at room temperature. The test solutions were prepared with DI water and chemical reagents from Thermo Fisher Scientific. An AMETEK SI VersaSTAT 4 Potentiostat Galvanostat was used for electrochemical testing. All experiments were carried out in a standard three electrode flat cell with an exposure area of 1 cm^2^ for the working electrode. Ag/AgCl and Pt mesh were used as reference and counter electrodes, respectively. All potential values are reported vs. Ag/AgCl (VAg/AgCl). Electrochemical tests were repeated at least three times to ensure the reproducibility of the results.

**REFERENCES:**

1. Persenot, T. *et al.* Effect of build orientation on the fatigue properties of as-built Electron Beam Melted Ti-6Al-4V alloy. *Int. J. Fatigue* **118**, 65–76 (2019).

2. Abramoff, M. D., Magalhães, P. J. & Ram, S. J. Image Processing with ImageJ. *Biophotonics Int.* **11**, 36–42 (2004).
